# Trend Analysis of Multidrug-Resistant Bacterial Pathogens Causing Neonatal Sepsis at University of Gondar Comprehensive Specialized Hospital, Northwest Ethiopia: A Retrospective Study

**DOI:** 10.1155/2021/9992994

**Published:** 2021-09-29

**Authors:** Mohabaw Jemal, Fetene Tinshku, Yeshwas Nigussie, Birhanetinsae Kefyalew, Chalie Alemu, Martha Belay, Teshome Belachew, Birhanu Ayelegn

**Affiliations:** ^1^University of Gondar, College of Medicine and Health Sciences, School of Biomedical and Laboratory Sciences, Department of Medical Microbiology, Gondar, Ethiopia; ^2^University of Gondar, College of Medicine and Health Sciences, School of Biomedical and Laboratory Sciences, Gondar, Ethiopia; ^3^University of Gondar, College of Medicine and Health Sciences, School of Biomedical and Laboratory Sciences, Department of Immunology and Molecular Biology, Gondar, Ethiopia

## Abstract

**Background:**

Neonatal sepsis is the most common cause of mortality and morbidity. It is a major global public health challenge, particularly in developing countries. Therefore, knowing the current status of bacterial isolates and their antimicrobial resistance profile is essential to physicians and health workers to implement appropriate intervention. The aims of this study was to assess a ten-year trend of bacterial prevalence isolates from blood culture among neonates (<1 month of age).

**Method:**

A hospital-based retrospective study was conducted on 1854 neonatal patients who were admitted at University of Gondar Specialized Comprehensive Hospital between 2010 and 2020. Sociodemographical and laboratory data were collected from medical records. Quality of the data was assured through standard operational procedures. Data were entered and analysed using SPSS version 20. Bivariate analysis was employed to determine strings of association between the outcome variable and sociodemographic variables. A *P* value less than 0.05 will be considered to be statically significant.

**Results:**

In a total of 1854 patients, 538 (29%) were culture positive. The overall neonatal sepsis infection rate was 287 (53.5%) for male and 249 (46.5%) for female. The highest proportion of neonatal sepsis infection rate was observed among the patients in the age range between 3 and 28 days and gestational at birth <37 weeks, 461(86%) and 278 (52%), respectively. Gestational at birth (*P* ≤ 0.001, AOR = 5.81, CI: 4.63–7.29) is significantly associated with bacterial isolates. The predominant pathogens were *Staphylococcus aureus,* 18 (76.6%), *Klebsiella pneumoniae,* 146 (38%), and *E*. *coli,* 45 (11.7%) among the age range less than one weak. *Klebsiella* spp, *S*. *aureus*, *and E*. *coli* showed a high level of resistance to most tested antimicrobials. Amikacin, ciprofloxacin and norfloxacin, and erythromycin were the most effective antibiotics whereas ampicillin, amoxicillin, and cotrimoxazole were the least effective antibiotics for isolates.

**Conclusion:**

Neonatal sepsis infection is common in the 3–28 days of age range. *S*. *aureus*, *E*. *coli,* and *K*. *pneumonia* were the most common isolates. Most the bacterial pathogens were resistant to commonly prescribed antibiotics. Therefore, an antimicrobial sensitivity test for bacterial isolates is recommended to provide updated data for the physician in choosing the appropriate antibiotic for better patient treatment outcome.

## 1. Background

Neonatal sepsis is defined as a systemic infection with bacteria, virus, and fungi with/without signs and symptoms of infection during the first 28 days of life. Sepsis can be categorizing as early onset (prior 72 hr in the life of the neonate) and late onset if the diagnosis was carried out after this deadline. Usually, early-onset neonatal sepsis is associated with perinatal background and late-onset neonatal sepsis is mainly related to the medical and surgical invasive procedures required by neonates who already have the disease. Early-onset sepsis remains a common and serious problem for neonates, especially preterm infants [1,2].

Neonatal sepsis is one of the major public health challenges. Based on different studies of different concerns, 41% (3.6 million) of all deaths in children under 5 years of age is due to bacterial infection. The majority of these deaths occur in low-income countries. Furthermore, neonatal mortality for different African countries ranges from 68 per 1000 live births in Liberia to 11 per 1000 live births in South Africa [3–5].

Existing published data have suggested that neonates are at the highest risk for bacterial sepsis, with the prevalence at 1 to 10 per 1000 live births worldwide [5]. The main bacterial pathogens of neonatal sepsis are *L*. *monocytogenes*, *Streptococcus*, *E*. *faecalis*, *E*. *faecium*, group D streptococci, *α*-hemolytic streptococci and staphylococci, *S. pneumoniae*, and *H. influenzae* type B, which are recognized as the principal cause of early neonatal sepsis. Among Gram-negative enteric organisms, predominantly, *E*. *coli*, *Klebsiella* species, and *N. meningitides* and *N. gonorrhea* have been also reported as a cause of neonatal septicemia [6, 7]. Bacteria such as staphylococci species and *E.coli* are predominantly caused late-onset sepsis [7]. Based on the current study, the prevalence of neonatal sepsis is increased during recent years, and it may be due to the development of resistant organisms [5]. Drug resistance of pathogens is a major problem in causing neonatal sepsis, especially in developing countries because of poor sanitary conditions, over-the-counter sale of antibiotics, and lack of legislation [5, 7]. Especially, MDR Gram-negative organisms, methicillin-resistant *S*. *aureus* (MRSA), and extended-spectrum beta-lactamase- (ESBL-) producing bacteria represent the principal setbacks to fight against infections. Most Gram-negative bacteria are now resistant to ampicillin and cloxacillin, and many are becoming resistant to gentamicin [7, 8]. The prevalence of bacterial organisms causing neonatal sepsis is constantly increasing, especially in developing and low-income countries, and the emergence of resistant bacteria has complicated the problem further. Therefore, this study aimed to assess ten-year trend of bacterial pathogens and their antimicrobial susceptibility pattern in neonatal patients with sepsis.

## 2. Materials and Methods

### 2.1. Study Design, Period, and Area

The study was conducted at University of Gondar Specialized Comprehensive Referral Hospital. It is found in the historical town of Gondar located 750 km far from the northwest of Addis Ababa, the capital city of Ethiopia, and in the North Gondar Zone of the Amhara National Regional State which is a metropolitan city in the Amhara National Regional State in Ethiopia. The hospital was purposely selected because that it is one of the biggest referral hospitals in the region. Currently, it provides services to more than five million people in Gondar town and the surrounding area. The hospital has an inpatient and outpatient department and laboratory service with more than 500 beds that provides health services such as surgery, internal medicine, pathology, TB/HIV, dermatology, antenatal care, delivery, postnatal care, laboratory, pharmacy, maternal and neonatal care, and other services for the population of Gondar town and surrounding areas [9].

### 2.2. Study Design, Period, and Data Collection

A retrospective cross-sectional study to assess the prevalence and antimicrobial resistance profile of neonatal septicemia was conducted from 2010 to 2020 at University of Gondar Specialized Comprehensive Referral Hospital, Gondar. Data records of 1854 neonatal patients were collected and reviewed using a checklist from medical microbiology laboratory registration logbooks. Sociodemographical data, laboratory test results, and hospitalization status were collected using a data extraction checklist.

### 2.3. Laboratory Methods

#### 2.3.1. Sample Collection and Processing

Two bottles of venous blood samples (1 ml for each bottle) from two different sites of the peripheral vein were collected aseptically by experienced nurses before any antibiotic use. The collected blood sample was inoculated into a Trypton soya broth blood culture medium pair of bottles (Becton, Dickinson, USA) under strict aseptic procedures [10]. All culture bottles were incubated aerobically at 37°C for seven days for the presence of visible microbial growth. Positive culture bottles were subcultured; subcultures were made during successive days on enriched and selective media including blood agar plate (Oxoid LTD), chocolate agar plate (incubated at 5% CO_2_ atmosphere), MacConkey (Oxoid Ltd., Basingstoke, Hampshire, UK), and mannitol salt agar plates and examined for growth after 24–48 h of incubation. All positive blood cultures were identified using their cultural characteristics, appearance on their respective media, and Gram staining reaction. Bacterial isolates were confirmed by the pattern of standard biochemical reactions. Gram-negative bacteria were identified by an oxidase test, indole production, citrate utilization, motility, urease, oxidase, gas production, hydrogen sulfide production, carbohydrate fermentation, and lysine decarboxylases production tests [11]. Identification of Gram-positive bacteria isolates: coagulase, catalase test and mannitol salt agar, bacitracin (0.04 or 0.05 UI), and optochin (5 *μ*g) susceptibility tests were conducted. Interpretation was made as sensitive if the zone diameter was at least 10 mm and resistant if the zone of diameter was less than 10 mm. Blood culture broth which shows no microbial growth within 7 days was reported as culture negative, only after results were subcultured on blood agar, Mac Conkey, and chocolate agar [11].

#### 2.3.2. Antimicrobial Susceptibility Tests

An antimicrobial susceptibility test was carried out for each identified bacterial isolates by using the disc diffusion method on Muller Hinton agar for nonfastidious organisms and Muller Hinton agar with 5% defibrinated sterile sheep blood for fastidious organisms [12]. Three to five pure colonies were emulsifies in 2 ml of nutrient broth. The bacterial suspensions' turbidity was matched and checked with 0.5 McFarland standards. Then, a sterile cotton swab was dipped into the suspension and squeezed free from excess fluid against the side of test tube. The test organisms were uniformly seeded on the surface of Muller Hinton agar and exposed to a concentration gradient of antibiotic diffusing from the antibiotic-impregnated paper disc into the agar medium, and the medium was incubated at 35°C for 18–24 h [13]. The following antimicrobial disks were used (Oxoid UK): amoxicillin-clavulanate (AMC:20/10 *μ*g), penicillin (P:10 unit), trimethoprim-sulfamethoxazole (SXT:1.25/23.75 *μ*g), erythromycin (E:15 *μ*g), tetracycline (TE:30 *μ*g), clindamycin (DA:2 *μ*g), chloramphenicol (CAF:30 *μ*g), ampicillin (AMP:10 *μ*g), gentamicin (CN:10 *μ*g), amikacin (AK:30 *μ*g), ciprofloxacin (CIP: 5 *μ*g), ceftazidime (CAZ: 30 *μ*g), ceftriaxone (CRO:30 *μ*g), and cefoxitin (CXT:30 *μ*g). Grades of susceptibility pattern were interpreted by comparison of the zone of inhibition according to the Clinical and Laboratory Standard Institute 2014 guideline [12].

#### 2.3.3. Quality Control

All media were prepared according to the manufacturer's instruction and following the standard operational procedure. Culture media were checked for sterility by incubating 5% batch of the media at 37^o^c for 24 hours, and performance of all prepared media was also checked by inoculating international standard-strains such as *E*.*coli* (ATCC 25922) for Gram-negative bacteria, *S*. *aureus* (ATCC 25923) for Gram-positive bacteria, and *N*. *Gonorrhoeae* (ATCC49226) for fastidious bacteria. To standardize the density of the inoculum of bacterial suspension for the susceptibility test, 0.5 McFarland standards were used.

#### 2.3.4. Data Management and Statistical Analysis

Data were entered and analysed by using SPSS version 20 software. The characteristics of the study population were summarized using frequencies, percentages, median, and standard deviation. Text descriptions, tables, and figures were used to describe or present the findings of the study. A chi-square test was used to compare the proportion of bacterial isolates with patients' age and year. A *P* value of <0.05 was considered statistically significant.

## 3. Results

### 3.1. Demographic Characteristics of Study Participants

Within the ten years, 1854 neonatal patients' data were collected and analysed at University of Gondar Specialized Comprehensive Referral Hospital. The majority, 995(53.7%), of the study participants were males. Out of the total, 1531(82.6%) of the study participants were 3–28 days old. The majority, 1425(76.9%), of neonates come from the NICU ward; of them, 27.8% were culture positive. The majority of bacterial isolates were observed between 2017 and 2019 (Table 1).

### 3.2. Prevalence of Neonatal Septicemia and Risk Factor

From the total 1854 blood samples analysed, 536/1854 (28.9%) were blood culture positive. Among the total numbers of bacterial isolates, 154 (28.7%) were Gram-positive isolates; of them, *S*. *aureus* were (76.6%) with the highest value followed by *Streptococcus viridians* (11%). Out of the total number of bacterial isolates, 384(71.3%) were Gram-negative bacteria. Among the predominant Gram-negative bacteria isolates, 146(38%) were *K*. *pneumoniae* followed by *K*. *ozaenae,* 47(12.2%), and *Enterobacter* species, 22(5.2%) (Table 2).

The predominant organisms were observed between 3 and 28 days of life, 461(86%), and the prevalence of bacteria isolates was observed in 0–3 days of life, 14 (75%). It is significantly associated with bacterial isolates (*P* ≤ 0.007, AOR = 1.52, CI: 1.12–2.02). The other risk factor associated with neonatal septicemia was gestational age at birth <37 weeks (*P* ≤ 0.007, AOR = 1.52, CI: 1.12–2.02), and 52% of preterm were blood culture positive. A majority of bacterial isolates were from neonatal patients who were admitted in the NICU, 396(74%) (Table 3).

### 3.3. Trends of Neonatal Septicemia

**Figure 1:**
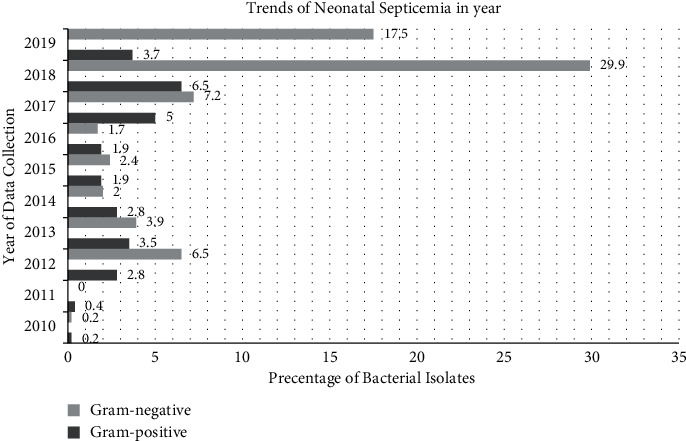
Trends of neonatal septicemia in years at University of Gondar Comprehensive Specialized Hospital, Gondar, Northwest Ethiopia, 2021.

The trends of bacterial isolates that caused neonatal sepsis among neonatal patients tend to decrease in years. The prevalence of neonatal septicemia significantly increased in 2017 (7.2% and 5%), 2018 (29.9% and 6.5%), and 2019 (17.5% and 3.7%), Gram-negative and Gram-positive bacterial isolates, respectively. On the other hand, the lowest prevalence of neonatal septicemia was in 2010 (0.2% and 0.2%) and 2011 (0% and 0.4%), Gram-negative and Gram-positive bacterial isolates, respectively (Figure 1).

### 3.4. Antibiotic Susceptibility Patterns of Bacterial Isolates from Neonatal Septicemia

Gram-positive bacterial species are also common causative agents for neonatal sepsis infection, significantly showing a high level of resistance to different antimicrobial agents. For instance, in this study, *S*. *aureus* and *CoNs* were the most predominantly isolated Gram-positive bacteria and had a great extent of resistance to ampicillin (83%), amoxicillin (87%), trimethoprim-sulfamethoxazole (81%), and norfloxacin (80%). On the contrary, amikacin (100%) and vancomycin (86%) were active antimicrobial agents against *S*. *aureus and CoNs*. On other hand, *S*. *pyogenes* were highly resistant to amoxicillin (100%) (Table 4).

Gram-negative bacterial species resistance to commonly used antimicrobials is alarmingly increasing. *Klebsiella* spp. and *E*. *coli* showed a high level of resistance to most tested antimicrobials. Ampicillin, amoxicillin, gentamycin, and ceftriaxone were among the tested antimicrobials resisted by *K*. *pneumoniae* with the resistance rate of 100%, 96%, 72%, and 90%, respectively. *E*. *coli* also showed a significant level of resistance to ampicillin (96%), and ceftriaxone (67%). However, antimicrobials such as kanamycin (89%) and norfloxacin (67%) had a good inhibition effect against *K*. *pneumoniae* (Table 5).

### 3.5. Multidrug-Resistant Isolates

Out of the total numbers of bacterial isolates, 76% were resistant to three or more different classes of antibiotics. Among multidrug resistant (MDR), 103(25.4%), 69(17%), and 163(40%) isolates were resistant to three classes of antibiotics, greater than four and five antibiotics, respectively. Results of drug resistance patterns compared within species specific showed that 80.4% of *S*. *aureus*, 40% of *K*. *pneumoniae*, and 10.2% of *E*. *coli* were MDR isolates (Table 6).

## 4. Discussion

Neonatal septicemia, a life-threatening worldwide disease, has become a disease of neonates less than one month of age with a significant mortality rate. It has to be reviewed periodically because of specific microorganisms responsible for infection varying with time, geography, and patient age. Out of the total culture, positive rate of bacterial isolates identified from patients with symptoms of neonatal septicemia was 29%, and this prevalence rate is higher than that reported from Nepal (12.6% and 10.8%) and Botswana (9.8%) [14–16]. But, it is lower than that of a study conducted in Egypt (40.7%), Sudan (61.3%), Yemen (57%), and in the Oromia region, southeast Ethiopia (34%) [17–20]. The variation could be due to fact that methodological variation, antibiotic administration before sample collection, control mechanisms in spread of bacterial infection, and difference in study setting might affect blood culture positivity rate.

In the present study, the prevalence of bacterial isolates was higher in males, 53.5%, than in females, 46.5%, which was comparable with the findings of previous studies where the prevalence of neonatal septicemia was higher in males (66%) than in females (33.9%) [16]. This might be due to the physiological and immunological makeup of nature and cultural practices in different societies.

The prevalence of neonatal septicemia was higher in the preterm (<37 weeks) than in gestational age >37 weeks (term). Similarly, studies in Ethiopia, Mexico, and Indonesia have also showed that preterm (<37 week) had a significant association with neonatal sepsis [2, 7, 21, 22]. This could be because a baby less than 37 weeks of age may have a limited capacity to increase neutrophil production in accordance to demand to overcome. In the current study, the predominant isolates causing neonatal septicemia from Gram-positive and -negative bacteria were *S*. *aureus*, 118 (76.6%), *K*. *pneumoniae*, 146(38%), and *CoNs,* 12(7.8%), respectively. Similarly, previous studies report that those isolates were also the major pathogens of neonatal septicemia [7, 18, 23].

In our study, amikacin, ciprofloxacin, and norfloxacin were the most effective drugs for Gram-negative isolates and vancomycin and ciprofloxacin were for Gram-positive isolates which correlates with the study conducted in Ethiopia [7]. *S*. *aureus* and *CoNs* showed the highest susceptibility towards vancomycin and ciprofloxacin. Amikacin showed 89% sensitivity towards *K*. *pneumoniae* isolates, similar to the findings of Muley et al. [24].

In this study, MDR was observed in *S*. *aureus* (80.4%), *K*. *pneumoniae* (40.5%), and *E*. *coli* (10.2%). Similarly, previous studies report that those MDR pathogens were the most common causative agents of neonatal septicemia [7, 25]. *S*. *aureus* and *K. pneumoniae* were common pathogens that showed MDR due to genetic competence to acquire antibiotic resistance genes from other strains, intrinsic resistance mechanisms, and chromosomal and plasmid-encoded beta-lactam hydrolyzing enzymes [7, 26, 27].

## 5. Conclusions and Recommendations

The prevalence of septicemia among neonatal patients was 29%. Gestational age at birth <37 weeks (preterm) is a high-risk factor for neonatal septicemia than gestational age at birth >37 weeks (term), and it is significantly associated with bacterial isolates (*P* ≤ 0.001, AOR = 5.81, CI: 4.63–7.29). Also, most of the bacterial pathogens were resistant to commonly prescribed antibiotics. Therefore, infection-preventive measures are required with a particular focus on neonatal patients. Further research is needed to explore the epidemiology and risk factors of neonatal septicemia.

## Figures and Tables

**Table 1:** Frequency of study participants by age groups, gender, year, and wards at University of Gondar Comprehensive Specialized Hospital, Gondar, Northwest Ethiopia, 2021.

Characteristics		Frequency	Percentage
*Sociodemographical*			
Sex	Male	995	53.7
	Female	859	47.3
Age group	0–3 days	323	17.4
	3–28 days	1531	82.6
Gestational age at birth	Preterm (<37 weeks)	488	26.3
	Term (>37 weeks)	1366	73.7
Admitted wards	NICU	1425	76.9
	N-wards	283	15.3
	Pediatrics	61	3.3
	Others	85	4.6

*Year of data collection*			
2010	Male	2	0.1
	Female	1	0.1
2011	Male	1	0.1
	Female	3	0.2
2012	Male	69	3.7
	Female	78	4.2
2013	Male	50	2.7
	Female	66	3.6
2014	Male	39	2.1
	Female	40	2.2
2015	Male	28	1.5
	Female	30	1.6
2016	Male	30	1.6
	Female	28	1.5
2017	Male	106	5.7
	Female	73	3.9
2018	Male	401	21.6
	Female	313	16.9
2019	Male	269	14.5
	Female	227	12.2

NICU: neonatal intensive care unit; others: emergency, gynecology, oncology, and medical wards.

**Table 2:** Percentage and frequency of bacterial isolates among neonates having sepsis at University of Gondar Comprehensive Specialized Referral Hospital, Gondar, Northwest Ethiopia, 2021.

Bacterial isolates	Frequency	Percentage
*Gram positive (n* *=* *154)*		
*S*. *aureus*	118	76.6
*S*. *viridans*	17	11
*Enterococcus* spp.	4	2.6
*S*. *pyogenes*	3	1.94
*CoNs*	12	7.8

*Gram negative (n* *=* *384)*		
*Enterobacter* spp.	22	5.7
*K*. *pneumoniae*	146	38
*K*. *rhinoscleromatis*	12	3.1
*Pseudomonas* spp.	2	18.8
*E*. *coli*	45	11.7
*NLFGNR*	41	10.7
*LFGNR*	29	7.5
*P*. *vulgaris*	3	0.8
*Serratia* spp.	4	1
*K*. *ozaenae*	47	12.2
*Citrobacter* spp.	33	8.6

NLGNR: nonlactose fermenter Gram-negative rods.

**Table 3:** Bivariate and multivariate analysis relationship between risk factors and bacterial isolates among neonates having sepsis at University of Gondar Comprehensive Specialized Referral Hospital, Gondar, Northwest Ethiopia, 2021.

Characteristics	Pos., *N*(%)	Neg., *N*(%)	COR (95% CI)	*P* value	AOR (95% CI)	*P* value
*Sex*						
Male	287(53.5)	708(53.7)	0.99(0.81–1.22)	0.946	1.02(0.82–1.26)	0.883
Female	249(46.5)	610(46.3)	1		1	

*Age group*						
0–3 days	75(14)	248(19)	1		1	
3–28 days	461(86)	1070(81)	1.42(1.08–1.89)	0.013	1.52(1.12–2.05)	0.007

*Gestational age at birth*						
Preterm (<37 weeks)	278(52)	210(16)	5.7(4.54–7.11)	≤0.001	5.81(4.63–7.29)	≤0.001
Term (>37 weeks)	258(48)	1108(84)	1		1	

*Admitted wards*						
NICU	396(74)	1029(78)	0.64(0.37–1.08)	0.094	0.63(0.36–1.13)	0.121
N-wards	91(17)	192(14.6)	0.78(0.44–1.39)	0.404	0.92(0.49–1.72)	0.795
Pediatrics	23(4.3)	38(3)	1		1	
Others	26(6)	59(4.4)	0.37(0.73–0.36)	0.370	0.80(0.38–1.69)	0.562

COR, crude odds ratio; AOR, adjusted odds ratio; CI, confidence interval.

**Table 4:** Antimicrobial susceptibility profile of Gram-positive bacterial isolates at University of Gondar Comprehensive Specialized Hospital, Northwest Ethiopia.

Gram positive	AMP, *N*(%)	AMX, *N*(%)	AMK, *N*(%)	CAF, *N*(%)	SXT, *N*(%)	CIP, *N*(%)	VAN, *N*(%)	CN, *N*(%)	CRO, *N*(%)	E, *N*(%)	OX, *N*(%)	NOR, *N*(%)	CXT, *N*(%)	DA, *N*(%)
*S*. *aureus*														
S	6(17)	3(13)	6(100)	25(46)	8(19)	33(49)	48(86)	10(50)	15(35)	16(22)	6(32)	4(20)	10(50)	33(25)
R	30(83)	20(87)	0(0)	30(54)	34(81)	34(51)	8(14)	10(50)	28(65)	57(78)	13(68)	16(80)	10(50)	13(75)

*S*. *viridans*														
S	0(0)	1(100)	1(100)	3(60)	0(0)	3(60)	8(67)	0(0)	1(20)	3(25)	2(67)	N/A	N/A	7(64)
R	2(100)	0(0.0)	0(0)	2(40)	7(100)	2(40)	4(33)	2(100)	4(80)	9(75)	1(33)	N/A	N/A	4(36)

*Enterococus* spp.														
S	0(0)	0(0)	N/A	N/A	1(100)	0(0)	1(100)	2(100)	0(0)	2(100)	0(0)	1(100)	N/A	0(0)
R	1(100)	1(100)	N/A	N/A	0(0)	1(100)	0(0)	0(0)	1(100)	0(0)	1(100)	0(0)	N/A	2(100)

*S*. *pyogenes*														
S	1(100)	1(33)	N/A	1(100)	N/A	0(0)	2(100)	0(0)	N/A	1(50)	N/A	N/A)	N/A	1(100)
R	0(0)	2(67)	N/A	0(0)	N/A	1(100)	0(0)	1(100)	N/A	1(50)	N/A	(N/A)	N/A	0(0)

*CoNs*														
S	0(0)	1(25)	1(100)	2(100)	1(33)	2(50)	10(91)	1(50)	0(0)	4(40)	0(0)	1(50)	1(100)	6(75)
R	1(100)	3(75)	0(0)	0(0)	2(67)	2(50)	1(9)	1(50)	1(100)	6(60)	3(100)	1(50)	0(0)	2(25)

AMP: ampicillin, CAF: chloramphenicol, SXT: trimethoprim-sulfamethoxazole, CIP: ciprofloxacin, VAN: vancomycin, CN: gentamicin, CRO: ceftriaxone, E: erythromycin, OXA: oxacillin, CXT: cefoxitin, NOR: norfloxacin, DA: clindamycin.

**Table 5:** Antimicrobial susceptibility profile of Gram-negative bacterial isolates at University of Gondar Comprehensive Specialized Hospital, Northwest Ethiopia, 2021.

Gram negative	AMP	AMX	TOB	CAF	AMK	CIP	GEN	CRO	E	NOR	CTX	AMC	SXT
*Enterobacter* spp.	*N*(%)	*N*(%)	*N*(%)	*N*(%)	*N*(%)	*N*(%)	*N*(%)	*N*(%)	*N*(%)	*N*(%)	*N*(%)	*N*(%)	*N*(%)
S	0(0)	1(7)	5(28)	6(35)	15(94)	18(69)	7(29)	6(32)	1(33)	8(73)	2(50)	0(0)	3(21)
R	17(100)	14(93)	13(72)	11(65)	1(6)	8(31)	17(71)	13(68)	2(67)	3(27)	2(50)	10(100)	11(79)

*K. pneumoniae*													
S	0(0)	1(4)	26(36)	20(49)	55(89)	72(64)	27(28)	6(10)	2(67)	8(67)	2(22)	1(9)	2(3)
R	69(100)	23(96)	47(64)	21(51)	7(11)	40(36)	70(72)	54(90)	1(33)	4(33)	7(78)	40(91)	75(97)

*K*. *rhinoscleromatis*													
S	0(0)	0(0)	3(43)	1(50)	3(75)	7(88)	2(25)	1(33)	0(0)	1(50	0(0)	0(0)	3(33)
R	8(100)	2(100)	4(57)	1(50)	1(25)	1(12)	6(75)	2(67)	1(100)	1(50)	0(0)	4(100)	6(67)

*E*. *coli *													
S	1(4)	1(5)	10(56)	7(58)	10(100)	22(79)	15(63)	4(33)	N/A	10(77)	4(67)	3(33)	6(29)
R	25(96)	20(95)	8(44)	5(42)	0(0)	6(21)	9(37)	8(67)	N/A	3(23)	2(33)	6(67)	15(71)

*NLFGNR*													
S	1(5)	1(9)	6(43)	7(58)	13(87)	26(84)	11(39)	6(27)	0(0)	4(50)	1(50)	1(100)	4(20)
R	20(95)	10(91)	8(57)	5(42)	2(13)	5(16)	17(61)	16(73)	2(100)	4(50)	1(50)	0(0)	16(80)

*LFGNR*													
S	0(0)	1(13)	7(58)	3(33)	8(80)	21(95)	8(42)	3(33)	N/A	N/A	1(50)	0(0)	3(18)
R	18(100)	7(87)	5(42)	6(67)	2(20)	1(5)	11(58)	9(67)	N/A	N/A	1(50)	1(100)	14(82)

*P*. *vulgaris*													
S	0(0)	0(0)	1(100)	N/A	1(50)	1(50)	1(50)	0(0)	N/A	N/A	2(100)	0(0)	N/A
R	1(100)	1(100)	0(0)	N/A	1(50)	1(50)	1(50)	1(100)	N/A	N/A	0(0)	1(100)	N/A

*Citrobacter* spp.													
S	0(0)	0(0)	2(17)	5(36)	11(100)	14(78)	4(21)	0(0)	1(50)	6(67)	1(25)	0(0)	4(22)
R	22100)	9(100)	10(83)	9(64)	0(0)	4(22)	15(79)	9(100)	1(50)	3(33)	2(75)	1(100)	14(78)

*Serratia* spp.													
S	0(0)	0(0)	N/A	1(50)	1(100)	3(100)	1(50)	N/A	N/A	N/A	2(100)	0(0)	2(100)
R	4(100)	2(100)	N/A	1(50)	0(0)	0(0)	1(50)	N/A	N/A	N/A	0(0)	1(100)	0(0)

*K*. *ozaenae*													
S	0(0)	0(0)	3(18)	3(21)	17(89)	21(66)	5(17)	3(12)	N/A	6(86)	N/A	2(15.4)	4(16)
R	29(100)	9(100)	14(82)	11(79)	2(11)	11(34)	25(83)	22(88)	N/A	1(14)	N/A	11(84.6)	21(84)

AMK- amikacin, AMP- ampicillin, NOR- norfloxacin, CRO- ceftriaxone, CAF- chloramphenicol, CIP- ciprofloxacin, E- erythromycin, GN- gentamycin, AMC- amoxicillin-clavulanate, TOB- tobramycin, SXT- trimethoprim-sulfamethoxazole, NLGNR- nonlactose fermenter Gram-negative rods.

**Table 6:** Multidrug-resistant patterns for Gram-positive and Gram-negative bacterial isolates at University of Gondar Comprehensive Specialized Hospital, Northwest Ethiopia, 2021.

Bacterial isolates	R3, N(%)	R4, N(%)	≥ R5, N(%)	Total
*Gram positive (n* *=* *112)*				
*S*.*aureus*	21(18.8)	22(19.6)	47(42)	90(80.4)
*S*. *viridans*	3(2.7)	5(4.5)	3(3.7)	11(9.8)
*Enterococcus* spp.	1(0.9)	2(1.8)	1(0.9)	4(3.6)
*S*. *pyogenes*	0(0)	0(0)	1(0.9)	1(0.9)
*CoNs*	2(1.8)	3(3.7)	1(0.9)	6(5.3)

*Gram negative (n* *=* *294)*				
*Enterobacter* spp.	4(1.4)	3(1)	13(4.4)	20(6.8)
*K*.*pneumoniae*	27(9.2)	43(14.6)	49(16.7)	119(40.5)
*K*. *rhinoscleromatis*	1(0.3)	2(0.7)	6(2)	9(3)
*Pseudomonas* spp.	1(0.3)	0(0)	0(0)	1(0.3)
*E*. *coli*	9(3)	9(3)	12(4)	30(10.2)
*NLFGNR*	9(3)	5(1.7)	12(4)	26(8.8)
*LFGNR*	11(3.7)	3(1)	8(2.7)	22(7.5)
*P*. *vulgaris*	0(0)	1(0.3)	0(0)	1(0.3)
*Serratia* spp.	0(0)	1(0.3)	1(0.3)	2(0.7)
*K*. *ozaenae*	9(3)	11(3.7)	17(5.8)	37(12.6)
*Citrobacter* spp.	5(1.7)	8(2.7)	14(4.8)	27(9.2)
Overall (*n* = 406)	103(25.4)	69(17)	163(40)	406(76)

R3, resistance to 3 drug classes; R4, resistance to 4 drug classes; ≥5, resistance to greater than 5 drug classes.

## Data Availability

Data and supporting materials associated with this study will be shared upon request.

## Abbreviations

*CoNs*: Coagulase-negative *Staphylococcus* species
CSF: Cerebrospinal fluid
ESBL: Extended-spectrum beta-lactamase
MDR: Multidrug resistant
MRSA: Methicillin-resistant *Staphylococcus aureus*
NICU: Neonatal intensive care unit
AOR: Adjusted odds ratio
EONS: Early-onset neonatal sepsis
EOS: Early-onset sepsis.
